# Human Induced Pluripotent Stem Cells Develop Teratoma More Efficiently and Faster Than Human Embryonic Stem Cells Regardless the Site of Injection

**DOI:** 10.1002/stem.471

**Published:** 2010-09

**Authors:** Ivan Gutierrez-Aranda, Veronica Ramos-Mejia, Clara Bueno, Martin Munoz-Lopez, Pedro J Real, Angela Mácia, Laura Sanchez, Gertrudis Ligero, Jose L Garcia-Parez, Pablo Menendez

**Affiliations:** Andalusian Stem Cell Bank, Centro de Investigación BiomédicaCSJA-UGR. Granada, Spain

Human embryonic stem cell (hESC) and reprogrammed/induced pluripotent stem cell (iPSC) research is becoming the “flavor of the month” for downstream applications such as drug screening, disease modeling, and future regenerative medicine and cell therapies [1–4]. Pluripotency (the ability to give rise to any cell type of the three germ layers: mesoderm, ectoderm, and endoderm) is the defining feature of hESCs and iPSCs [5]. In vivo teratoma formation in immune-compromised mice is the “gold-standard” assay to define bona fide pluripotent stem cells capable of generating tumoral disorganized structures containing tissues representing the three germ layers [5,6]. Despite the importance of teratoma assay as an extended screen for the pluripotency of hESCs and iPSCs and as in vivo assay to explore molecular and cellular mechanisms underlying the biology of human teratomas and their transition to teratocarcinomas, there are no standard procedures for performing this assay [5–7]. Different studies on hESCs have correlated the site of implantation with the efficiency of teratoma formation and histology tissue composition [6,8]. However, limited data are available regarding the teratoma development latency. More importantly, no study so far has compared side-by-side the efficiency, latency, and histological tumor composition of hESCs- and iPSCs-derived teratomas. In addition, a new generation of immunodeficient mice has been developed: the NOD/SCID IL2Rγ^−/−^ mouse. This strain carries a IL2Rγ-chain deficiency that blocks signaling through multiple cytokine receptors leading to many innate immune defects [9,10]. The non obese diabetic/severe combined immune-deficient (NOD/SCID) IL2Rγ^−/−^ strain facilitates engraftment and tumor formation and does not develop thymic lymphoma, ensuring a longer lifespan of inoculated mice.

Here, we followed the improved teratoma protocol previously developed by Prokhorova et al. [6,11–13] to transplant side-by-side as few as 1 × 10^6^ of either fully characterized undifferentiated hESCs or iPSCs in 6- to 8-week-old non obese diabetic/severe combined immune-deficient (NOD/SCID) IL2Rγ^−/−^ mice [11,13–15]. The following hESC lines were used: H9, H1, AND1, AND2, AND3, HS181, and ECAT. The following iPSC lines were used: MSHU-001, iAND4, CB-CD34+ iPSC1, and CB-CD34+ iPSC2. These lines have been fully characterized and deposited according to Spanish Legislation at The Spanish Stem Cell Bank (http://www.isciii.es/htdocs/terapia/terapia_lineas.jsp) [16]. Briefly, cells were resuspended in phosphate buffered saline (PBS) supplemented with 30% matrigel (Becton Dickinson, San Jose, CA, http://www.bd.com) [6] and transplanted subcutaneously (200 μl volume) or by intratesticular injection (60 μl volume). Figure 1A depicts the experimental strategy used. We then analyzed efficiency, latency, and histological tumor composition. In hESCs, the rate of teratoma formation was 81% subcutaneously versus 94% intratesticularly (*n* = 30 mice; Fig. 1B). However, the intratesticular injection, despite showing higher efficiency of teratoma formation, displayed a slightly longer latency (66 vs. 59 days; *p*-value > 0.05). There were no site-specific differences in the teratoma composition at the histological level (Fig. 1C). Interestingly, when iPSCs were transplanted the rate of teratoma formation was 100% (*n* = 16 mice), regardless the type of injection. More importantly, iPSCs seem more aggressive in vivo as the latency was shortened 52% (from 59 days to 31 days) upon subcutaneous injection and 26% (from 66 days to 49 days) upon intratesticular injection. As with hESCs, no differences in teratoma composition were observed either.

**Figure 1:**
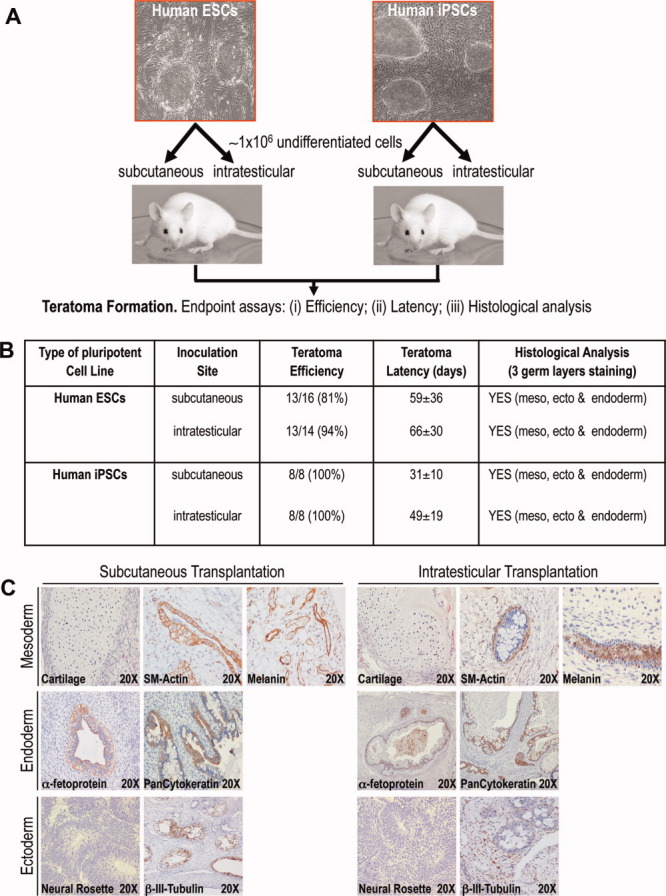
Human iPSCs form teratomas faster and with higher efficiency than hESCs regardless the site of injection. (**A**): Cartoon summarizing the experimental design. (**B**): Table summarizing the efficiency, latency, and histological analysis of the teratomas developed from both human ESCs and human iPSCs upon either subcutaneous or intratesticular transplantation. (**C**): Histological analysis of teratomas derived from both hESCs and iPSCs upon subcutaneous or intratesticular transplantation reveal similar tissue composition. The presence of mesodermal tissues is defined by teratoma sections containing cartilage and positive for both smooth muscle actin and melanin. The presence of ectodermal tissues was scored as teratoma sections that stained positive for β-III tubulin and presence of neural rosettes. Endoderm was defined as teratoma sections that stained positive for α-fetoprotein and pan-cytokeratin. The study was approved by the Andalusian and Spanish Embryo and Cellular Reprogramming Ethical Institutional review Board. Animal protocols were approved by the Local University Hospital Council On Animal Care and Experimentation. Abbreviations: ESCs, embryonic stem cells; iPSCs, reprogrammed/induced pluripotent stem cells.

To the best of our knowledge, this is the first study comparing side-by-side the efficiency, latency, and teratoma composition between hESCs and iPSCs. We found clear differences in the efficiency and latency but not in the teratoma histological composition. Further experiments are still demanded to gain insights into the higher aggressiveness in vivo of iPSCs as compared with hESCs. Ploidy, analyzed by conventional G-banding karyotype, could not explained these differences because all but two pluripotent stem cell lines were euploid: the aneuploid lines were one hESC (AND1) and one iPSC (iAND4). It is worth emphasizing, however, that karyotype analysis is not a high-resolution technique detecting fine genomic aberrations, with a euploid karyotype not being therefore indicative of an overall cellular genomic stability. Whether or not specific tiny genomic insults (detectable by high-resolution methods such as comparative genomic hybridazation (CGH)-arrays and single-nucleotide polymorphism analysis) or epigenetic differences may explain the higher aggressiveness in vivo of iPSCs still needs to be elucidated. We envision that these data may be useful not only for stem cells scientists addressing pluripotency issues and studying mechanisms underlying specific germ-layer/tissue differentiation but also for cancer researchers developing in vivo models for germ cell tumors.

## Disclosure of Potential Conflicts of Interest

The authors indicate no potential conflict of interest.
